# Improved Therapeutic Efficacy of Doxorubicin Chemotherapy With Cannabidiol in 4T1 Mice Breast Cancer Model

**DOI:** 10.1002/cam4.70395

**Published:** 2024-11-06

**Authors:** Koorosh Tabatabaei, Sara Moazzezi, Mohammadreza Emamgholizadeh, Haleh Vaez, Behzad Baradaran, Behrooz Shokouhi

**Affiliations:** ^1^ Student Research Committee Tabriz University of Medical Sciences Tabriz Iran; ^2^ Faculty of Veterinary Medicine Tabriz Islamic Azad University Tabriz Iran; ^3^ Department of Pharmacology and Toxicology, Faculty of Pharmacy Tabriz University of Medical Sciences Tabriz Iran; ^4^ Immunology Research Center Tabriz University of Medical Sciences Tabriz Iran; ^5^ Department of Pathology Tabriz University of Medical Sciences Tabriz Iran

**Keywords:** breast cancer, cannabidiol, cardiotoxicity, doxorubicin, lung metastasis

## Abstract

**Background:**

High dose chemotherapy is one of the therapeutic strategies for breast cancer and doxorubicin (DOX) as a chemotherapy agent is widely used. DOX indication is limited due to its dose‐depended cardiotoxicity. Recently, cannabidiol (CBD) shows antitumoral and cardioprotective effects, so we hypothesized that CBD administration with high‐dose DOX chemotherapy can improve anticancer activity and reduce cardiotoxic side effects.

**Method:**

Mice breast cancer model established by injecting 4T1 cell lines. One group was not injected by 4T1 cells as a not cancerous group and received normal saline (NS, 0.1 mL). In cancerous groups, first group was considered as cancerous control and received NS (0.1 mL); the second group received CBD (5 mg/kg, IP) on Days 1,7, and 14; in the third group DOX (5 mg/kg, IV) as CBD schedule was administrated; the fourth group treated with CBD 1 day before DOX injection as pretreatment, and the last group was treated with CBD and DOX at same time with previous doses and schedules. On Day 21, all mice were sacrificed, heart and lungs tissues were obtained and histological sections were isolated. SOD2, iNOS, MMP2, MMP9 were evaluated through western blot and TUNEL test preformed for breast tumor.

**Results:**

Tumor size and weight significantly decreased in DOX, pretreatment CBD + DOX and CBD + DOX groups. Administration of CBD with DOX could not prevent weight loss. TUNEL test demonstrated the highest tumor cell apoptosis in pretreatment CBD + DOX and CBD + DOX. In lungs belonged to CBD + DOX, there was not any sign of metastasis. Cardiac histopathological examination of pretreatment CBD + DOX and CBD + DOX did not show any sign of congestion or inflammation. In CBD + DOX SOD2 increased, also iNOS, MMP2, and MMP9 decreased compared to DOX.

**Conclusions:**

This study demonstrated that simultaneous administration of CBD and DOX can increase antitumoral effect and reduce DOX cardiotoxicity. Nevertheless, CBD can induce cardiotoxicity as administrated alone.

## Graphical abstract

Cannabidiol administration at same time or as pretreatment, can increase antitumoral effect of doxorubicin CBD administration at same time with DOX can reduce DOX cardiotoxicity unlike pretreatment therapy. CBD alone had a cardioroxic effect.

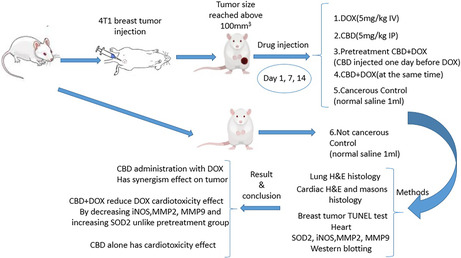

**Abbreviations**
AKT: Protein kinase B
BAX: Bcl‐2‐associated X protein
BLBC: basal‐like breast cancer
CBD: Cannabidiol
DOX: Doxorubicin
dUTP: deoxy uridine phosphate
ECL: Enhanced Chemiluminescence
GPR55: G protein‐coupled receptor 55
H&E: Hematoxylin and Eosin
ID‐1: inhibitor of differentiation 1
iNOS: Inducible nitric oxide synthase
MMP2: Matrix metalloproteinase‐2
MMP9: Matrix metalloproteinase‐9
MTD: Maximum tolerated dose
mTOR: Mammalian target of rapamycin
MTT: Meaning–text theory
NSCLS: Non–small cell lung cancer
PBS: Phosphate buffered saline
RIPA: Radio‐immunoprecipitation assay
ROS: Reactive oxygen species
RPMI: Roswell Park Memorial Institute
SOD2: Superoxide dismutase‐2
TBS‐T: Tris buffered saline with tween
TV: Tumor volume

## 1 Introduction

Breast cancer is one of the most frequently diagnosed malignancies worldwide specially in women [1, 2]. Although scientific and technological advances have progressively improved patient survival rates and enabled better detection and clinical management of the disease, breast cancer mortality remains the second highest among cancers in women. It is resulted in approximately 290,000 deaths annually worldwide, with over 40,000 deaths in the United States [3].

Breast cancer mortality is mostly due to tumor metastasis [4]. Lungs are one of the most common sites of breast cancer metastasis and present a major clinical challenge, as patients' median survival after diagnosis is less than 2 years [5]. Notably, lung metastasis usually occurs within 5 years of preliminary breast cancer diagnosis and significantly impacts patient survival. Intractable lung metastases remains the cause of 60%–70% of death in breast cancer [6]. Patients with metastasis limited solely to the lung, have a very poor prognosis, with a median survival of only 25 months [7]. This poor outcome is related to narrow range of treatment associated with inoperable lesions [4]. Although various approaches developed to cancer treatment, systemic chemotherapy remains the principal cancer therapy. Pharmacological progress in the late 20th century, increased the life expectancy of cancer patients [8].

One chemotherapy strategy is high‐dose chemotherapy, which has been successful in some cancers, such as testicular cancer, and has shown acceptable survival rates [9]. However, it should be noted that under the conditions of successful cancer treatment with chemotherapeutic drugs, complications related to toxic effects on healthy tissues and organs have come to the fore, so these side effects in using of high‐level chemotherapy should be considered [10]. However, despite improvement in cancer treatment, complications associated with adverse effect on healthy tissues and organs step forward. Anthracycline cardiomyopathy was the first serious cardiovascular complication to have been noticed by oncologists and cardiologists worldwide [11].

Doxorubicin is a subordinate metabolite of mutant strains of streptomyces peucetius var. caesius and categorized in the anthracycline family. Doxorubicin is used as an effective antitumor agent against several types of cancer [12]. Its anticancer activity is primarily mediated by DNA intercalation and the inhibition of topoisomerase II enzymes in rapidly growing tumors. Diverse acute or chronic side effects such as cardiotoxicity may limit the use of doxorubicin in a dose‐dependent manner [13]. Approximately, 4%–26% of patients receiving doxorubicin resulted in myocardial dysfunction, whereas higher doses lead to a prevalence of 18%–26%. Similarly higher risk of cardiotoxicity as 36%–48% belongs to doses above 600 mg/m^2^ [14]. Anthracycline‐associated cardiotoxicity is typically progressive and irreversible, leading to different pathological conditions of ventricular dysfunction, heart failure, and arrhythmias [15].

Considering its extended anticancer effectiveness, various strategies used to reduce its cardiotoxicity including structural remodeling and synthesis of doxorubicin analogs (such as epirubicin and idamycin) or novel formulation like liposomal doxorubicin; and combination of active ingredients by pharmacological approaches. The latter, which has been extensively studied, commonly uses cardioprotective agents [16]. The neuroregulatory network of endocannabinoid System “ECS” involved in the homeostasis of most systems in the body [17]. Agents interact in ECS are gaining attention as pharmacological approaches to counter cancer and improve the quality of life. In this regard, cannabinoids have been studied in many preclinical models as systemic therapeutic choice [18].

A wealth of data suggests that the anticancer effects of cannabinoids are exerted via different signaling mechanisms at multiple levels of tumor progression. Inhibition of tumor cell proliferation [19], tumor invasion [20] and metastasis [21], angiogenesis [22] and chemoresistance [23] and induction of apoptosis and autophagy are evidenced [24]. Various animal cancer models have demonstrated the preventive ability of cannabidiol in progression of different types of cancer, including glioma [24], breast [25], lung [19], prostate [26] and cervical [27] cancer. For instance, cannabidiol significantly reduced tumor size and increased survival in a melanoma mouse model [28].

There is increasing evidence that cannabidiol can act synergically with various chemotherapeutic agents to enhance their efficacy. A possible adjuvant role in combination with cisplatin is suggested as sensitizing the cancer cells to and significantly increasing cisplatin‐mediated apoptosis [29]. Accordingly, the endocannabinoid system draw attention as a new therapeutic option in different cardiovascular related diseases such as atherosclerosis, myocardial infarction, and heart failure [30]. Furthermore, it is reported that cannabidiol diminished oxidative heart damage in hypertension, diabetes, and chemotherapy‐induced cardiotoxic effects, which introduces it as a potential cardioprotective agent [31].

Due to importance of doxorubicin in cancer chemotherapy and its remarkable cardiotoxicity, on the other hand anticancer and cardioprotective potential of cannabidiol, in this study, we used cannabidiol simultaneously with doxorubicin and also as a pretreatment in a mouse model of breast cancer (4T1 cell line). For this purpose, the following factors have been measured:

TUNEL test for evaluation and confirmation of tumor apoptosis, lungs macroscopic and microscopy evaluation for tumor metastasis, histopathological examination of heart to evaluate cardiotoxicity. Also, SOD2 as an anti‐oxidant enzyme, iNOS as a major source of NO production, MMP2 and MMP9 for determine cardiac inflammation were assessed through western blotting.

## 2 Methods and Materials

### 2.1 Cell Culture

4T1 breast cancer cell line was purchased from the Hematology and Oncology research center of Tabriz, Iran. Cells were cultured in complete RPMI medium containing 10% fetal bovine serum, 5% amino acids and 5% antibiotics (penicillin and streptomycin) at 37°C containing CO_2_. All experiments were performed using a fresh cell line which preserved in liquid nitrogen.

### 2.2 Reagent

Doxorubicin (EBEDOXO, EBEWE Inc., Austria), liposomal CBD (INOVO LIPOMED, KMT Inc., Iran) were purchased. Mouse monoclonal antibodies to SOD2, iNOS, MMP2, MMP9 and β‐actin as primary anti bodies, m‐IgGκ BP‐HRP and mouse anti‐rabbit IgG‐HRP as secondary antibodies for western blot all purchased from SANTA CRUZ BIOTECHNOLOGY Inc., Oregon, USA. TUNEL assay kit (Elabscience Biotechnology Inc., Houston, USA) used for TUNEL test.

### 2.3 Animal 4T1 Breast Cancer Model

A total of 30 female Balb/c (6–8 weeks old and 16–22 g) mice purchased from Pasteur Institute laboratory animal laboratory (Tehran, Iran), and kept in standard conditions with free access to water, standard pellet and normal lighting. 4T1 breast tumors were established by injecting of 10^6^ tumor cells in lower mammary pad of each 25 mice and 5 mice were not injected as not cancerous control mice.

### 2.4 Experiment Condition

When the tumor reached above 100 mm^3^, all cancerous mice were randomly divided into five groups (*n* = 5). The first group was the control cancerous group, which received only saline (0.1 mL, IP) on Days 1,7 and 14. The second group administered only cannabidiol (5 mg/kg, IP) on Days 1, 7 and 14 [32]. The third group only administrated doxorubicin (5 mg/kg, IV) as cannabidiol schedule [33]. The fourth group received cannabidiol (5 mg/kg, IP) 1 day before doxorubicin (5 mg/kg, IV). The fifth group administered cannabidiol (CBD 5 mg/kg, IP) + doxorubicin (DOX 5 mg/kg, IV) on Days 1, 7, 14. The non‐cancerous (healthy) control group (*n* = 5) just received saline (0.1 mL, IP) on Days 1, 7 and 14. The treatment was continued for 21 days with weekly treatment schedule. Animal's weight and tumor volume were recorded twice a week. Tumor volume measured by caliper and calculated by the formula of tumor volume:

TV = 1/2 (length × width^2^).

On Day 21, all the mice were weighed again and sacrificed under anesthesia by ketamine (80 mg/kg) and xylazine (10 mg/kg). The heart tissues were isolated, weighed, washed with PBS, and then separated into two pieces, one for histopathological examination and the other one was stored at −80°C for western blotting assay. The tumor was isolated for TUNEL test and weighed. Also, the lung tissue was isolated to macroscopic and microscopic evaluation of metastasis. All animal procedures were performed in accordance with the Guide for the Care and Use of Laboratory Animals of Tabriz University of Medical Sciences and was approved by the Ethics Committee of Tabriz University of Medical Sciences (approval number: IR.TBZMED.VCR.REC.1400.518). In addition, the study was designed and implemented under the recommendations of the ARRIVE guidelines for reporting animal research [34].

### 2.5 TUNEL Assay and Tumor Apoptotic Index Evaluation

Apoptosis was examined by the Terminal deoxynucleotidyl transferase dUTP nick end labeling (TUNEL) kit (Elabscience Biotechnology Inc., Houston, USA) according to the manufacturer [35] Briefly after deparaffinization, sections were incubated with Proteinase k Tris/HCl, pH = 7.4–8 (working solution 10 Mm) for 30 min at 37°C. After the incubation period, the slices were washed two times with PBS. Then 50 μL TUNEL reaction mixtures were added and placed in the dark for 1 h at 37°C. Afterward, samples were analyzed in drop of PBS under a fluorescence microscopy (Olympus BX50) using excitation wavelength in the range of 450–500 nm. To evaluate tumors apoptotic index, green areas of TUNEL results were considered as apoptotic cells and these areas were measured and calculated by image J software version 1.53k.

### 2.6 Lungs Histopathological Examination

The lung tissues from each animal, were fixed in 10% formalin solution, dehydrated in ascending grades of alcohol and embedded in paraffin. The paraffin blocks were cut to thin sections using a microtome and stained with hematoxylin and eosin (H&E). The sections were examined under light microscope by a pathologist unaware of the treatment protocol.

### 2.7 The Heart Weigh to Body Weight Assessment

Isolated heart tissues were washed by cold saline, dried, and weighed. The wet heart weight to body weight ratios were calculated by this formula:

Heart weight/Body weight = heart weight (mg)/Body weight (g).

### 2.8 Heart Histopathological Examination

Isolated hearts were fixed in 10% formalin solution, dehydrated in ascending grades of alcohol and embedded in paraffin. The paraffin blocks were cut serially then stained with H&E and Masson's trichrome. The sections were examined under light microscope by a pathologist unaware of the treatment protocol.

### 2.9 Western Blot Assays

Western blot analysis were performed according to assay described by Chang G et al. [36] and Samarjit Das et al. [37] with minor modifications. Briefly, proteins were extracted from tissues using lysis buffer in the presence of proteinase cocktail. Protein extracts were subjected to 14% sodium dodecyl sulfate polyacrylamide gel electrophoresis and blotted into polyvinylidene fluoride membranes. After blocking with 5% non‐fat milk, membranes were incubated with primary antibodies against superoxide dismutase 2 (SOD2), inducible nitric oxide synthase (iNOS), matrix metalloproteinase 2 and 9 (MMP2 and MMP9) and anti‐β‐actin at 4°C about 18 h. Then all membranes washed three times with TBS‐T, secondary antibodies added and incubated 75 min at 25°C. Immunoblots were visualized using ECL reagents (Pierce, Rockford, IL, USA). Densitometric analysis of the immunoblots was performed using image j software (Wayne Rasband, National Institute of Health, USA).

### 2.10 Statistics

Data were presented as mean ± SEM. The data were statistically analyzed using one‐way ANOVA followed by LSD post hoc test to compare the mean values between the treatment groups and the control. Differences were considered significant at *p* < 0.05.

## 3 Result

### 3.1 Effects of CBD Administration With DOX Chemotherapy on Breast Tumor Size and Weight

**FIGURE 1:**
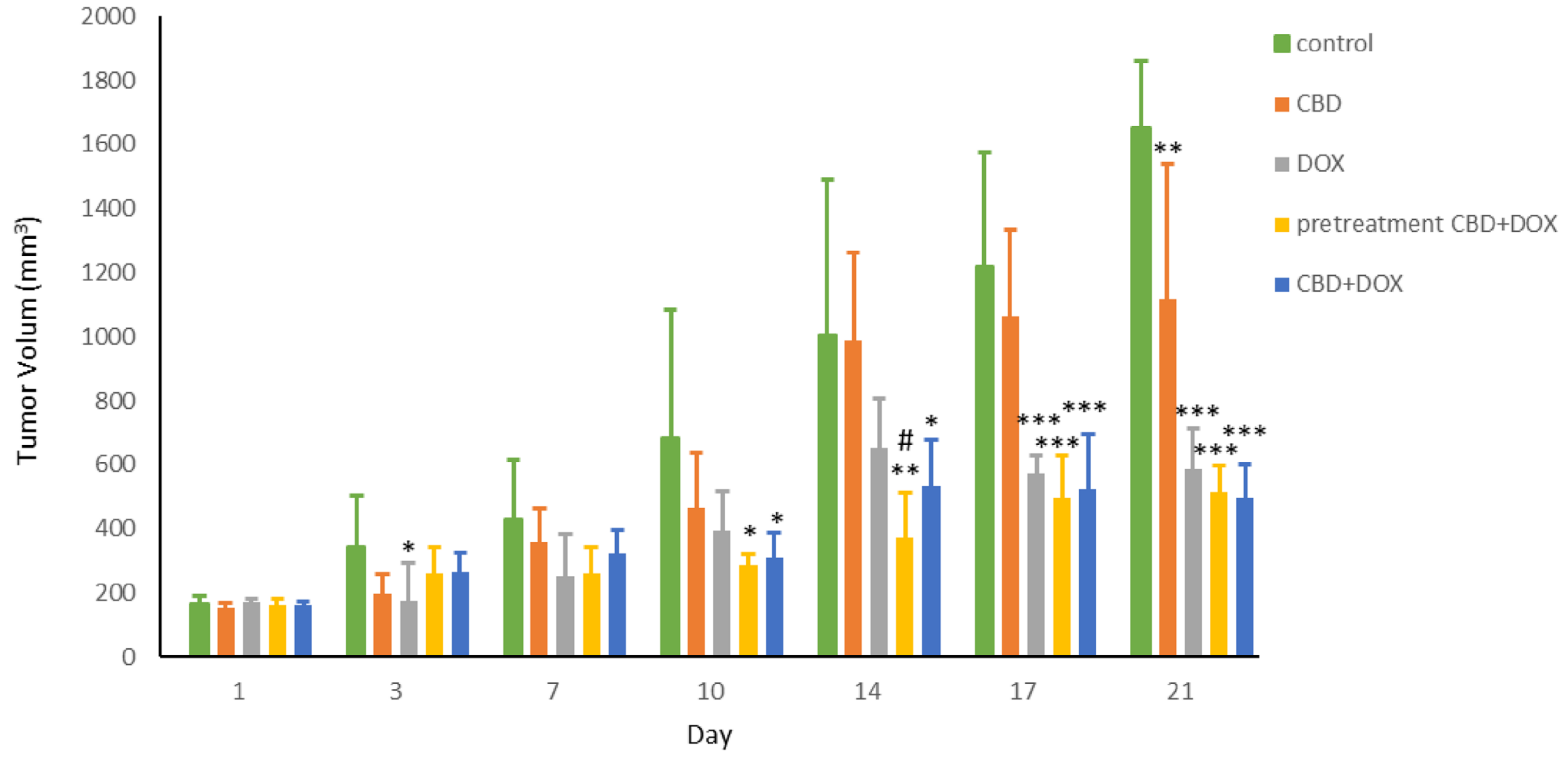
Effects of cannabidiol and doxorubicin on mean tumor size during 21 days of treatment. Results are presented as mean ± SEM (*n* = 5). CBD (5 mg/kg), DOX (5 mg/kg), pretreatment (CBD 5 mg/kg 1 day before DOX 5 mg/kg) and CBD + DOX (5 mg/kg) at same time compared to control group (normal saline). *p < 0.05, **p < 0.01, ***p < 0.001 from control group and ^#^ *p* < 0.05 from DOX group using one way ANOVA with LSD post hoc test.

**FIGURE 2:**
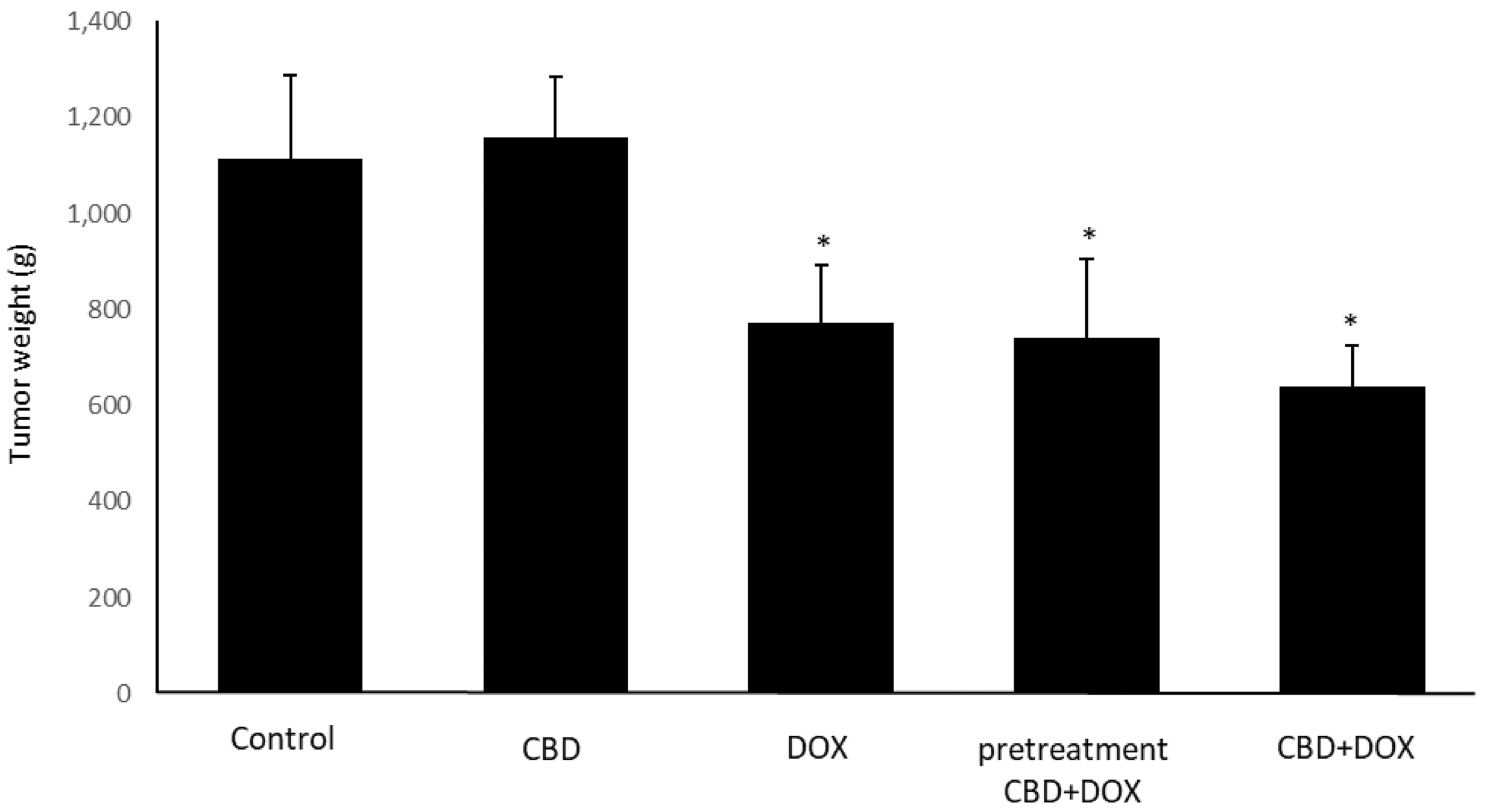
Effects of cannabidiol and doxorubicin on tumor weight. Results are presented as mean ± SEM (*n* = 5). CBD (5 mg/kg), DOX (5 mg/kg), pretreatment (CBD 5 mg/kg 1 day before DOX 5 mg/kg) and CBD + DOX (5 mg/kg) at same time compared to control group (normal saline). *p < 0.05 from control group and CBD group using one way ANOVA with LSD post hoc test.

According to the data measurement, administration of CBD + DOX at same time or as a pretreatment significantly shrank tumor size in these two groups like the doxorubicin group compared with the control group. On Day 14, pretreatment group has a better effect compared to doxorubicin group (Figure 1). Also, the evaluation of tumors weights after excision showed enormously drop in CBD + DOX, pretreatment CBD + DOX and the group only received doxorubicin in comparison to control group (Figure 2).

### 3.2 Effects of CBD Administration With DOX Chemotherapy on Weight Loss During Experiment

**FIGURE 3:**
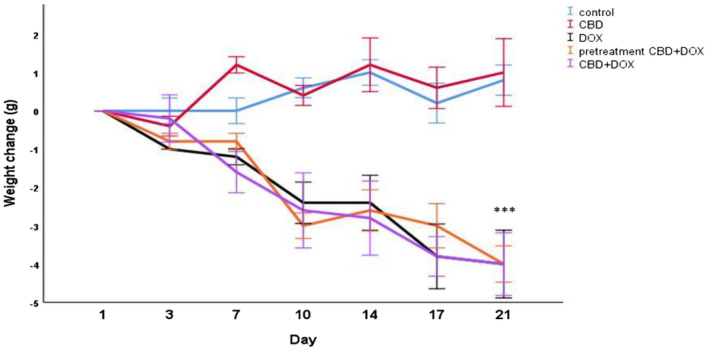
Body weight changes during experiment. Negative values indicate weight loss, whereas positive values represent weight gain. Results are presented as mean ± SEM (*n* = 5). ***p < 0.001 from control group using one way ANOVA with LSD post hoc test.

**FIGURE 4:**
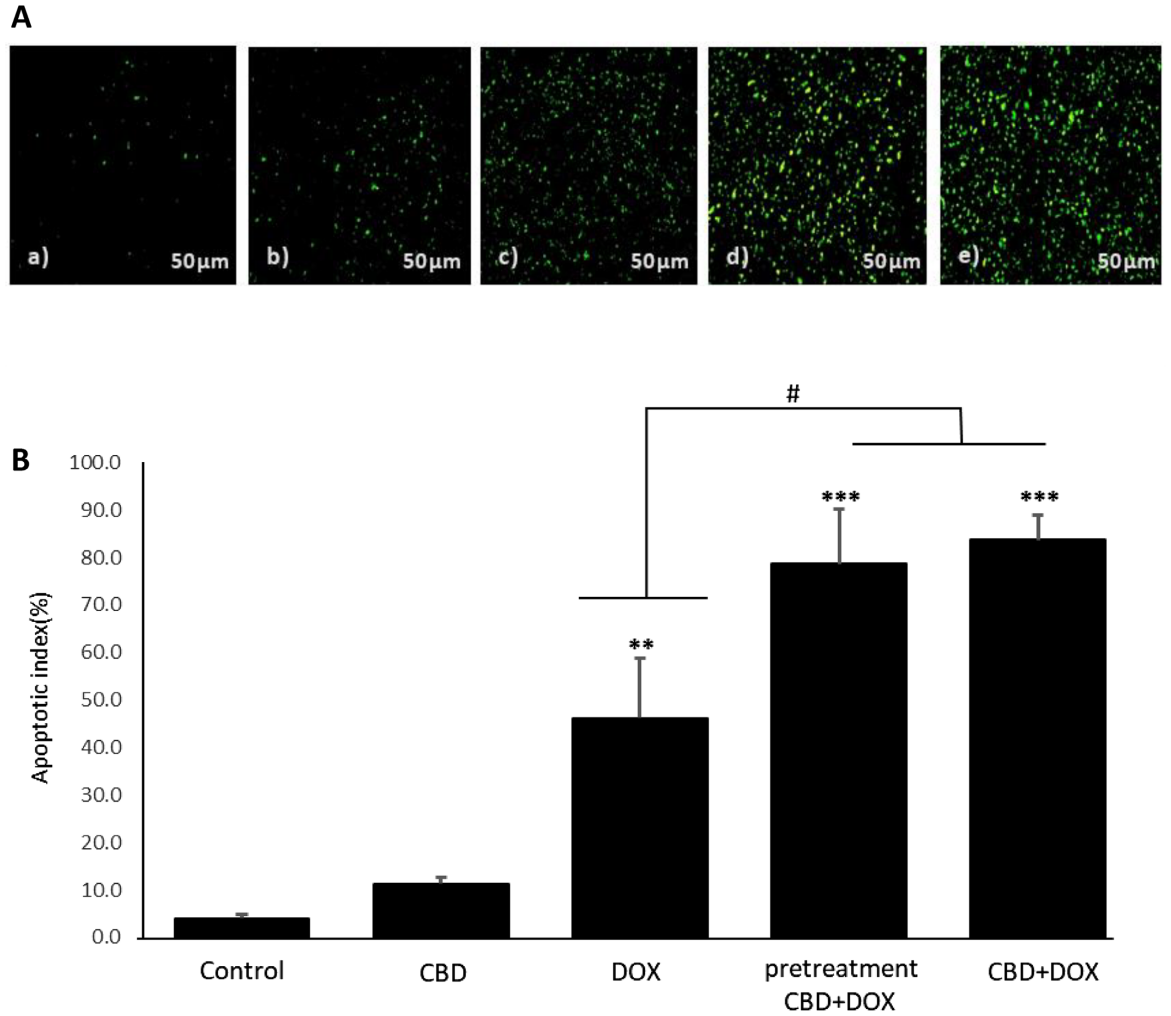
Effects of cannabidiol and doxorubicin on apoptosis in breast tumor. (A) TUNEL test for evaluation of apoptosis. (a) control, (b) CBD, (c) DOX, (d) pretreatment CBD + DOX, (e) CBD + DOX (*n* = 3, 450–500 nm). (B) Apoptotic index (percentage) of TUNEL test of breast tumor. Results are presented as mean ± SEM (*n* = 3). CBD (5 mg/kg), DOX (5 mg/kg), pretreatment (CBD 5 mg/kg 1 day before DOX 5 mg/kg) and CBD + DOX (5 mg/kg) at same time compare to control group (normal saline). **p < 0.01 and ***p < 0.001 from control group, #*p* < 0.05 from DOX group using one way ANOVA with LSD post hoc test.

To determine the toxicity of the treatment in this study, the average weight loss was measured. As it is demonstrated in Figure 3, in all doxorubicin receiving groups body weight dramatically decreased about 4 g during 21 days of treatment, which this weight loss is significant compared to control group (*p* < 0.001) and express the severe toxicity of doxorubicin. There was no significant weight change in control and cannabidiol group (Figure 4).

### 3.3 Effects of CBD Administration With DOX Chemotherapy on Tumor Apoptosis

**FIGURE 5:**
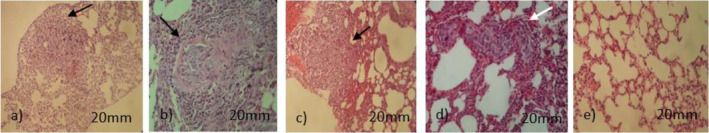
Histological appearance of lungs sections stained with hematoxylin and eosin. (Magnification of 20×, light microscopy) with metastasis (black arrows), (*n* = 5); (a) control (normal saline) showing large and sever metastasis with deformity, (b) CBD (5 mg/kg) like control group showing large sever metastasis and deformity, (c) DOX (5 mg/kg) showing metastasis but it is less than control and CBD groups, (d) pretreatment CBD + DOX showing intravascular metastasis (white arrow), (e) CBD + DOX showing lungs with almost normal appearance.

To evaluation whether cannabidiol with doxorubicin can trigger cell death responses in 4T1 breast tumor cells, TUNEL test was performed. The observation of DNA fragments in the TUNEL confirms the occurrence of apoptosis. As the result indicated CBD + DOX and pretreatment groups are significantly stained green fluorescent even more than doxorubicin group, that means successful induction of apoptosis in response to the cannabidiol addition to doxorubicin treatment (Figure 5a). Also, in quantification of these data, apoptotic index demonstrated in Figure 5b which shows that pretreatment group and CBD + DOX group have a great apoptotic index (*p* < 0.001) compared to control group and as we can see in pretreatment and CBD + DOX group, apoptotic index is significantly higher than doxorubicin group (*p* < 0.05).

### 3.4 Effects of CBD Administration With DOX Chemotherapy on Lung Metastasis

**FIGURE 6:**
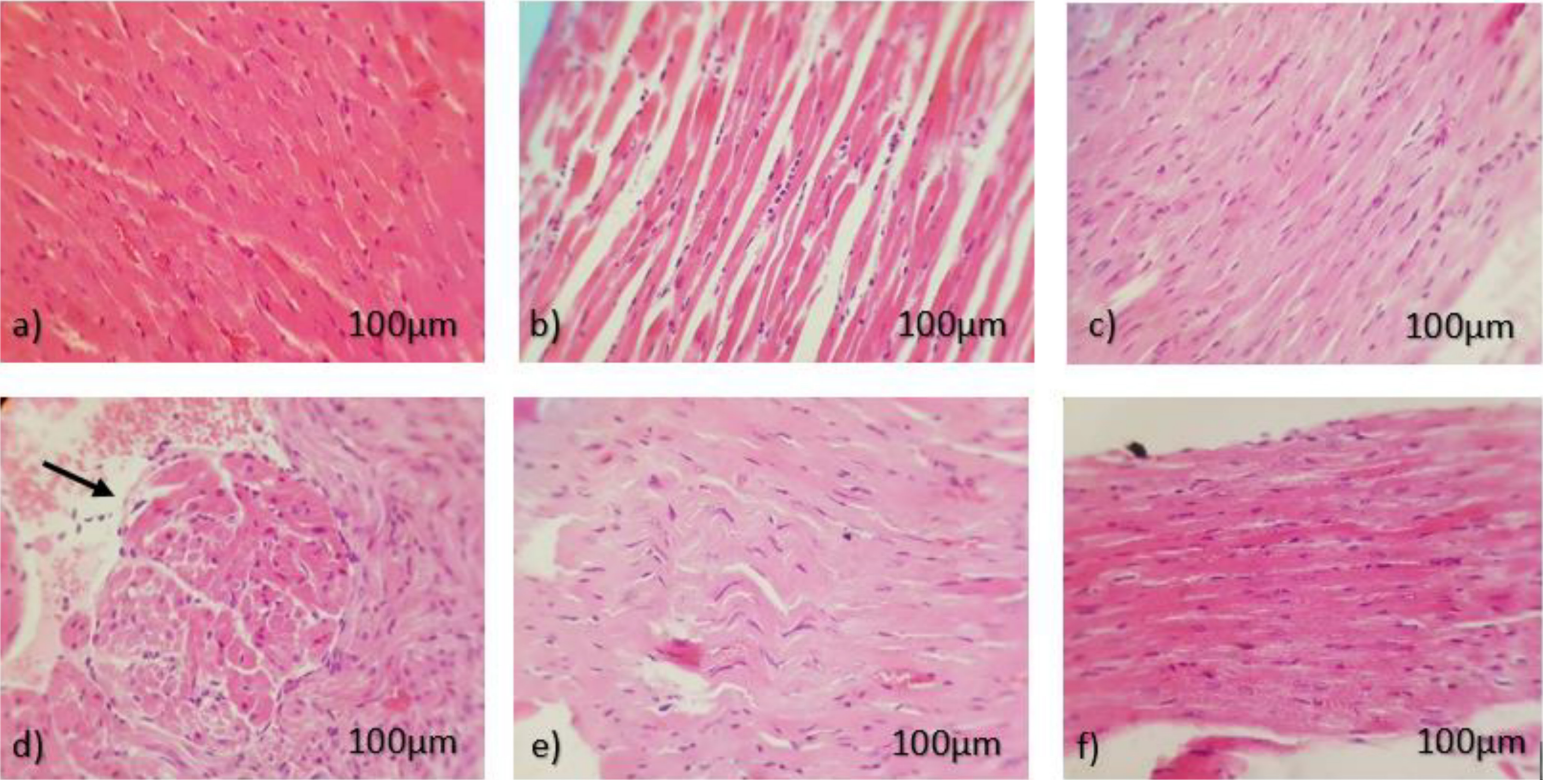
Histological appearance of heart sections stained with hematoxylin and eosin. (Magnification of 40×, light microscopy), (*n* = 5). (a) Non‐cancerous control (normal saline), (b) cancerous control (normal saline), (c) CBD (5 mg/kg), (d) DOX (5 mg/kg) showing mild inflammation (black arrow), (e) pretreatment CBD (5 mg/kg) 1 day before DOX (5 mg/kg), (f) CBD + DOX (both 5 mg/kg).

The lungs were evaluated macroscopic after euthanasia and microscopic in histopathology. There was no sign of any metastasis in CBD + DOX group but in other groups metastasis was observed. In pretreatment group, just in one of the lung samples an intravascular metastasis was found (Figure 6).

### 3.5 Effects of CBD Administration With DOX Chemotherapy on Heart Weight and Heart to Body Weight Ratio

The heart weight and heart weight to body weight (HW/BW) ratio were determined to assess the extent of edematous developed by doxorubicin and to answer this question whether treatment with cannabidiol may protect the heart from doxorubicin‐induced cardiac damages. It was observed that, there was not any significant difference between all groups in these parameters. However, heart weight and body weight decreased significantly in DOX, pretreatment CBD + DOX and CBD + DOX groups compared to cancerous control group (*p* < 0.001) (Table 1).

**TABLE 1:** Body and heart weight after 21 days experiment, heart weight divided to body weight has been calculated. Values are the mean ± SEM (*n* = 5).

List	Not cancerous control	Cancerous control	CBD	DOX	Pretreatment CBD + DOX	CBD + DOX
Body weight (g)	20.6 ± 1.1	22 ± 2.5	18.8 ± 1.6	16 ± 1.8***	15.8 ± 1.7***	16.2 ± 2.1***
Heart weight (mg)	92.8 ± 5.8	113.6 ± 34.5	92.4 ± 11	79.8 ± 10.3***	80.2 ± 8.4***	80.4 ± 7.5***
Heart weight/Body weight (mg/g)	4.5 ± 0.14	4.53 ± 0.23	5.14 ± 1.41	4.92 ± 0.49	5.06 ± 0.21	4.97 ± 0.13

*** *p* < 0.001 versus cancerous control by using on way ANOVA with LSD post hoc test.

### 3.6 Effects of CBD Administration With DOX Chemotherapy on Cardiac Histopathology

Histological evaluation by light microscopy demonstrated that in all groups except than doxorubicin group, the myofibrils were contiguously aligned and the morphology and the structure of the nuclei and cells were normal. Doxorubicin group demonstrated a diffuse and focal inflammation (Figure 3). Furthermore, Masson's trichrome staining was performed to assess the development of fibrosis in all groups; however no change was observed in any groups (Data are not shown).

### 3.7 Assessment of Cardiac Protein Levels Through Western Blotting

#### 3.7.1 Effects of CBD Administration With DOX Chemotherapy on Oxidative Stress and SOD2 Regulation

**FIGURE 7:**
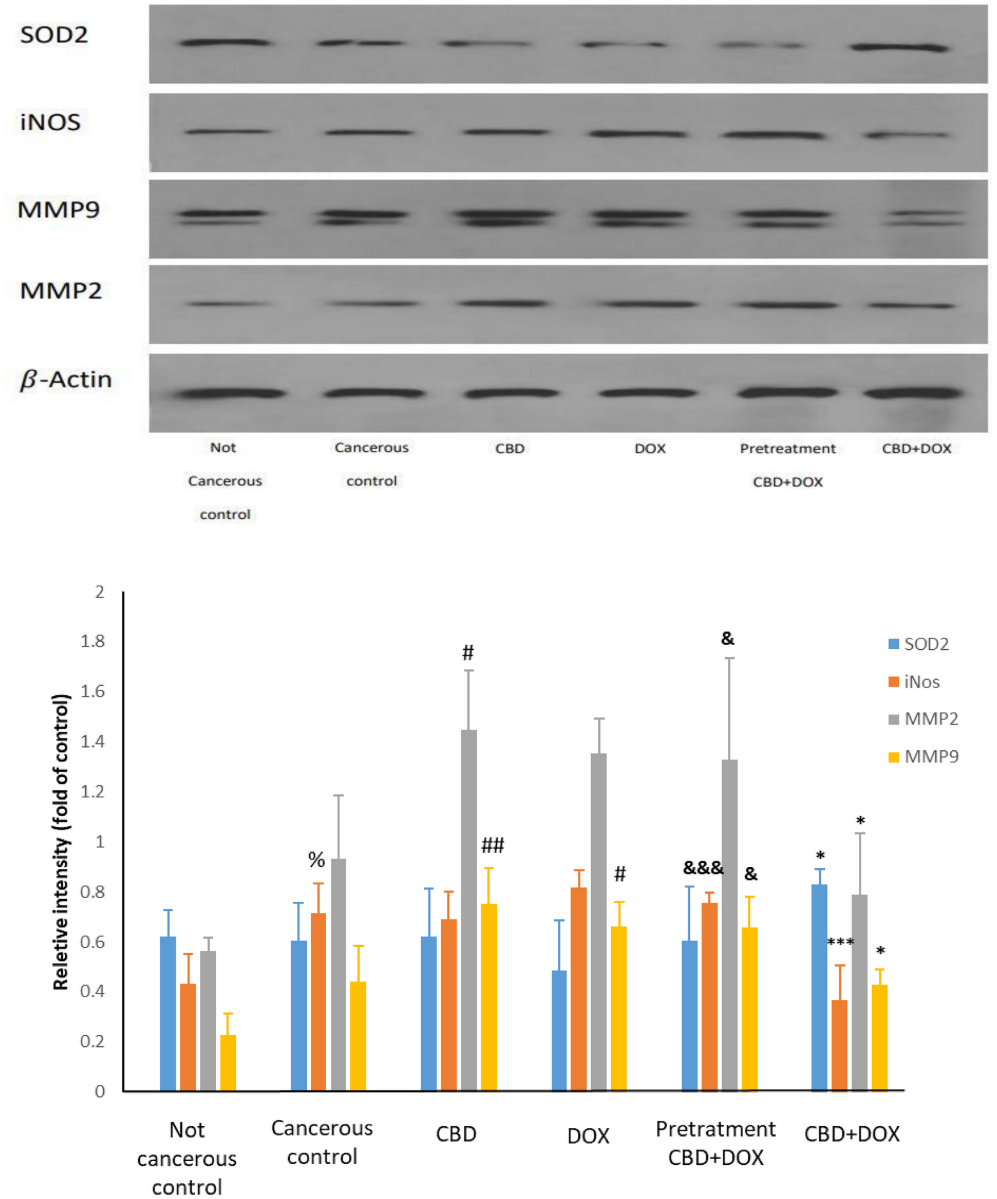
Effects of CBD and high‐dose DOX chemotherapy on heart proteins of SOD2, iNOS, MMP2, and MMP9. Results are presented as mean ± SEM (*n* = 3). *p < 0.05 and ***p < 0.001 versus DOX, ^#^ *p* < 0.05 and ^##^ *p* < 0.01 versus cancerous control, ^&^ *p* < 0.05 and ^&&&^ *p* < 0.001 versus CBD + DOX group, ^%^p<0.05 versus not cancerous control using one way ANOVA with LSD post hoc test.

We further investigated whether the addition of cannabidiol could activate SOD2 as an antioxidant in the myocardium. As shown in Figure 7 there was no difference between cancerous and not cancerous group. Also, there was not any significant change in cannabidiol and doxorubicin compared to cancerous control group. Nevertheless, cannabidiol administration with doxorubicin at same time can increase SOD2 significantly (*p* < 0.05), which surprisingly in the pretreatment group this change was not significant as like as cannabidiol group.

#### 3.7.2 Effects of CBD Administration With DOX Chemotherapy on iNOS Protein

Evaluation of iNOS as a major source of NO production was performed to examine cannabidiol potency with doxorubicin chemotherapy at same time or as pretreatment in reducing this protein. There was significant change in level of iNOS between cancerous and not cancerous control group (*p* < 0.05). Cannabidiol and doxorubicin did not show meaningful change compared to cancerous control group. While in contrast, cannabidiol administration with doxorubicin at same time reduced iNOS protein significantly (*p* < 0.001). Pretreatment group compared to CBD + DOX group has a higher expression of iNOS protein and there was not any significant difference in comparison to doxorubicin group. Thereby, it meant that, pretreatment therapy could not reduce this protein unlike CBD + DOX group.

#### 3.7.3 Effects of CBD Administration With DOX Chemotherapy on MMP2 and MMP9

To determine cardiac inflammation related protein expression, we measured MMP‐2 and MMP‐9 proteins. As demonstrated in Figure 7, these proteins levels were not changed between cancerous and not cancerous group. In doxorubicin and cannabidiol groups there were accumulation of these proteins, while, in CBD + DOX group this parameter was significantly lower compared to doxorubicin group (*p* < 0.05). The pretreatment group did not induce any significant changes in comparison to doxorubicin group, although it was significantly higher than CBD + DOX group (*p* < 0.05).

## 4 Discussion

Here, we presented that treatment with cannabidiol and high‐dose doxorubicin in mouse breast cancer model has a better antitumor effect and can reduce doxorubicin cardiotoxicity. As mentioned before breast cancer is the most common malignancy in the world and the leading cause of cancer deaths [1]. Distant metastasis accounts for the vast majority of deaths in patients with cancers like basal‐like breast cancer (BLBC), and displays a lung tropism of metastasis [4]. Therefore, effective treatment strategies are necessary to eradicate drug resistance, deliver a payload to the site of action in a tumor microenvironment and prevent tumor metastasis. Doxorubicin as an extensively used chemotherapeutic drug has several problems due to lack of selectivity [38, 39], which one of them is dose‐dependent cardiotoxic effects [40]. Despite the enormous number of researches conducted to reduce or prevent this toxicity [41, 42], cardiac adverse effect is still a potentially serious complication of high‐dose chemotherapy [10].

Cannabidiol (CBD) obtained from *Cannabis sativa* is able to interfere with different stages of the tumor process; it can inhibit cancer cell migrations and adhesions, and exerts anti‐proliferative, pro‐apoptotic, and anti‐invasive effects [43]. It is proposed that cannabidiol induces endoplasmic reticulum stress and apoptosis by inhibiting the AKT/mammalian target of rapamycin (mTOR) signaling and enhances reactive oxygen species (ROS) generation for selected breast cancer cells [44].

In this study tumor size and weight decreased in pretreatment and CBD + DOX group. Accordingly, TUNEL test demonstrated that co‐administration of cannabidiol and doxorubicin at the same time or as pretreatment could induce more apoptotic cells in breast tumors compared to doxorubicin or cannabidiol alone. Previous studies also reported significant synergistic interactions between cannabidiol and other chemotherapeutic drugs in breast cancer mcf7 cell line [45]. In vitro and in vivo study on MDA‐MB‐231 cells demonstrated that cannabidiol with doxorubicin can increase the expression of proteins involved in apoptosis like caspase‐3 and BAX; furthermore tumor size was significantly decreased [32] as observed in this study.

Result of lung histopathology did not show any sing of lung metastasis in CBD + DOX group. The evidences showed that, cannabidiol can suppress the growth and metastasis of cisplatin‐resistant non‐small cell lung cancer (NSCLC) [46]. Furthermore in colorectal cancer protective effect of cannabidiol against tumor invasiveness, migration and metastasis were reported [47]. Cannabidiol also inhibits the invasiveness of aggressive MDA‐MB‐231 and MDA‐MB‐436 breast cancer cell lines by downregulating inhibitor of DNA binding 1 (ID‐1), a transcriptional regulator, which stimulates the metastasis in a mouse model of advanced breast cancer with lung metastases [48]. Moreover other cannabidiol‐related studies reported inhibition of GPR55 expression, which is related directly or indirectly to cell changes promoting malignant growth, including uncontrolled cancer cell proliferation, angiogenesis, cancer cell adhesion, cancer cell migration, and metastasis [49]. We also demonstrated that in pretreatment CBD + DOX group, there was still sign of lung micro metastasis. May be cannabidiol injection with doxorubicin at the same time has a stronger effect in metastasis pathways compered to pretreatment therapy [50].

To provide information on major adverse effects of maximum tolerated dose (MTD), evaluation of parameters such as body weight loss and food consumption level besides clinical signs typically performed [51]. In other hand, weight loss is a common manifestation in patients with cancer [52]. In this study cannabidiol administration with doxorubicin at the same time or as a pretreatment model could not prevent weight loss during high‐dose chemotherapy. Although evidences suggest that cannabinoids are associated with reduced chemotherapy toxicity like improvement of nausea, vomiting and cachexia and also increase in appetite [53, 54], in this investigation cannabidiol could not prevent doxorubicin‐induced weight loss which may be resulted as high‐dose doxorubicin [33]. In the group just received cannabidiol, non‐significant weight gain compared to cancerous control group observed, that seems cannabidiol can increase appetite.

Although, body weight and heart weight decreased in DOX, pretreatment CBD + DOX and CBD + DOX group; heart weight to body weight ratio did not change significantly between groups. It seems in this study DOX did not cause a severe congestion in heart, and reduction in heart weight and body weight may relate to high dose DOX adverse effects as mentioned.

Histopathological studies in this experiment did not show any interstitial cardiac fibrosis or vascular fibrosis and myocardial cell count change in the doxorubicin group, which may be as a result of short‐term duration of study, and also maybe resulted because our cumulative dose didn't reach 20 mg/kg [55]. Nevertheless, signs of inflammation were observed as demonstrated in previous studies [56].

As it was indicated, high‐dose doxorubicin chemotherapy can cause cardiotoxicity through different pathways and its pathogenesis has traditionally been attributed to the formation of reactive oxygen species (ROS) [57]. Superoxide dismutases (SODs) are a class of enzymes that catalyze the detoxification of superoxide into oxygen and hydrogen peroxide, and then converted to oxygen and water by catalase [58]. SOD2 plays a crucial role in controlling ROS production [59]. Our results showed that coadministration of cannabidiol and doxorubicin, significantly increased SOD2 in accordance to previous evidences demonstrating antioxidant agents can reduce doxorubicin cardiotoxicity [60]. The previous data prove that cannabidiol has antioxidant activity [61], and also similar to our results, in disease like diabetes mellitus cardiomyopathy, cannabidiol considered as a cardioprotective agent [62] with decreasing the myocardial ROS generation and expression of p22phox, p67phox, gp91phox, restoring glutathione content, improving SOD activity and reducing 3‐NT formation. However, in the pretreatment protocol this impact diminished. It seems cannabidiol activate antioxidant system when ROS formation rises in the body and therefore preventive therapy before exposure could not activate this protective system.

Inducible nitric oxide (NO) synthase (iNOS) overexpression has been considered detrimental in heart failure, based on observations of NO‐mediated depression of cardiomyocyte contraction in human and mice studies [63]. Our results demonstrated that CBD + DOX reduced iNOS expression and enhanced cardiac function unlike the other groups. Doxorubicin enhanced NO formation, following the induction of iNOS, that has been implicated in the pathogenesis of inflammation and reduced cardiac function [64]. In cancerous control group, in overall, protein expression enhanced and iNOS increased significantly. This can be resulted as cancer cells metabolites related ROS production and oxidative stress induction in other cells and changes in protein expression [65]. In pretreatment condition, unlike the coadministration status, iNOS did not change significantly, which is assumed that maybe cannabidiol treatment prior to doxorubicin acts as like as administration of cannabidiol alone and could not reduce cardiotoxicity as mentioned in SOD2 explanation.

MMPs regulate the remodeling process by facilitating extracellular matrix turnover and inflammatory signaling, especially in cardiac remodeling [66]. In this study MMP2 and MMP9 level were significantly lower in CBD + DOX in contrast to doxorubicin group. Similar result also reported indicating reduced level of MMP9 and MMP2 mRNA by coadministration of cannabidiol and doxorubicin, besides doxorubicin‐induced increase in MMP9 and MMP2 level [55, 67].

As this study demonstrate, CBD alone increased MMP2 and MMP9, it seems CBD alone could cause mild inflammation in heart. Although there are not many evidences to demonstrate that CBD could induced cardiotoxicity, in few studies it was demonstrated chronic exposure of this drug could have some harmful effects in cardiovascular and reproductive systems [68, 69].

In overall, this study showed that CBD had cardioprotective effect when it used with DOX at same time. This investigation also demonstrates that perhaps CBD act as a double‐edged sword and this drug only effects as a cardioprotective agent when a detrimental process had been started in heart. Therefore, when CBD used as pretreatment, because DOX detrimental effect had not been started CBD did not act as a cardioprotective agent. Furthermore, it seems CBD started a detrimental process by increasing MMP2 and MMP9 in pretreatment group as CBD group.

It is suggested to examine the other pathways of cardio protective effects of cannabidiol against doxorubicin injuries, like endothelial activation signaling pathway (eNOS/AKT). Also, detrimental effects of cannabidiol alone in cancerous mice needs to be evaluated in detail.

## 5 Conclusion

This study demonstrated the potent efficacy of cannabidiol in mouse breast cancer model with high‐dose chemotherapy on the antitumor, anti‐metastasis and cardioprotective roles against doxorubicin. Simultaneous administration of cannabidiol with high‐dose doxorubicin not only improved the antitumor and anti‐metastasis efficacy, but also could reduce cardiotoxicity by decreasing MMP2 and MMP9 and improving cardiac function by decreasing iNOS. Furthermore, cannabidiol could improve antioxidant system by increasing SOD2. Eventually, these findings demonstrated cannabidiol as a potential effective agent in coadministration with doxorubicin at the same time in improving anticancer effects and reducing cardiotoxicity.

## Author Contributions

**Koorosh Tabatabaei:** conceptualization (lead), data curation (equal), formal analysis (equal), investigation (equal), methodology (equal), writing – original draft (equal), writing – review and editing (equal). **Sara Moazzezi:** data curation (equal), investigation (equal), methodology (equal), writing – original draft (equal), writing – review and editing (equal). **Mohammadreza Emamgholizadeh:** data curation (supporting), funding acquisition (supporting), investigation (supporting), project administration (supporting). **Haleh Vaez:** conceptualization (equal), data curation (equal), formal analysis (equal), funding acquisition (equal), investigation (equal), methodology (equal), project administration (lead), resources (equal), software (equal), supervision (lead), validation (equal), visualization (equal), writing – original draft (lead), writing – review and editing (lead). **Behzad Baradaran:** investigation (supporting), project administration (supporting), supervision (supporting), validation (supporting). **Behrooz Shokouhi:** data curation (supporting), investigation (supporting), methodology (supporting), validation (supporting), visualization (supporting).

## Ethics Statement

All animal procedures were performed in accordance with the Guide for the Care and Use of Laboratory Animals of Tabriz University of Medical Sciences and was approved by the Ethics Committee of Tabriz University Student Research Committee of Medical Sciences (Approval number: IR.TBZMED.VCR.REC.1400.518). In addition, the study was designed and implemented under the recommendations of the ARRIVE guidelines for reporting animal research.

## Conflicts of Interest

The authors declare no conflicts of interest.

## Data Availability

The original data supporting these findings are available at any time upon request to the corresponding author.
